# Linking Microbial Community Composition in Treated Wastewater with Water Quality in Distribution Systems and Subsequent Health Effects

**DOI:** 10.3390/microorganisms7120660

**Published:** 2019-12-07

**Authors:** Abasiofiok Mark Ibekwe, Shelton E. Murinda

**Affiliations:** 1 US Salinity Laboratory, USDA-ARS, 450 W. Big Springs Rd., Riverside, CA 92507, USA; 2 Animal and Veterinary Sciences Department, Center for Antimicrobial Research and Food Safety, California State Polytechnic University, Pomona, CA 91768, USA; semurinda@cpp.edu

**Keywords:** biofilm, pathogen, drinking water, water distribution systems, public health

## Abstract

The increases in per capita water consumption, coupled in part with global climate change have resulted in increased demands on available freshwater resources. Therefore, the availability of safe, pathogen-free drinking water is vital to public health. This need has resulted in global initiatives to develop sustainable urban water infrastructure for the treatment of wastewater for different purposes such as reuse water for irrigation, and advanced waste water purification systems for domestic water supply. In developed countries, most of the water goes through primary, secondary, and tertiary treatments combined with disinfectant, microfiltration (MF), reverse osmosis (RO), etc. to produce potable water. During this process the total bacterial load of the water at different stages of the treatment will decrease significantly from the source water. Microbial diversity and load may decrease by several orders of magnitude after microfiltration and reverse osmosis treatment and falling to almost non-detectable levels in some of the most managed wastewater treatment facilities. However, one thing in common with the different end users is that the water goes through massive distribution systems, and the pipes in the distribution lines may be contaminated with diverse microbes that inhabit these systems. In the main distribution lines, microbes survive within biofilms which may contain opportunistic pathogens. This review highlights the role of microbial community composition in the final effluent treated wastewater, biofilms formation in the distribution systems as the treated water goes through, and the subsequent health effects from potential pathogens associated with poorly treated water. We conclude by pointing out some basic steps that may be taken to reduce the accumulation of biofilms in the water distribution systems.

## 1. Introduction

‘Access to safe drinking-water is essential to health, a basic human right and a component of effective policy for health protection [1]’. In 2011, the World Health Organization (WHO) provided guidelines for drinking water quality and indicated that ‘water is essential to sustain life, and a satisfactory (adequate, safe, and accessible) supply must be available to all [1].’ They also noted that improving access to safe drinking water can result in tangible benefits to health, therefore, every effort should be made to provide drinking water that is as safe as practicable. The nature and form of drinking-water standards may vary among countries and regions due to the economic advantages of these countries and the available technologies in water treatment, such as, microfiltration and reverse osmosis after tertiary treatment. The essential requirements for safe drinking water must include health-based guidelines set by competent health authorities to properly manage the water infrastructure. These may include among other things infrastructure monitoring and independent surveillance [1]. WHO, therefore, emphasized the importance of securing the microbial safety of drinking-water based on the use of multiple barriers, starting from catchment to consumers? This organization noted that this action will prevent the contamination of drinking water or to reduce contamination to levels not injurious to health. Therefore, particular attention should be directed to implementing comprehensive water safety plans that would ensure drinking-water safety and protect public health [1]. 

In most municipalities, source water is treated and passed through different processes before they are available for potable use. During primary treatment, the incoming wastewater is channeled through screens to remove bulky, insoluble solids and the remaining colloidal matter can settle in a primary clarifier preceding sludge treatment. For instance, in some of the most advanced water treatment systems, undisinfected influent or secondary treated municipal wastewater may go through the purification process from microfiltration (MF), reverse osmosis (RO), and UV light. In some processes H_2_O_2_ is added before UV treatment as well as lime to further stabilize the purified water [2]. In most cases, these processes may reduce microbial population to an undetectable level, and at this point the water is ready to be acceptable for numerous applications, from irrigation and industrial processing to domestic water supply [3]. Orange County Water District’s groundwater replenishment system has one of the largest and most internationally recognized potable water recycling operations that currently serves 2.4 million residents in Orange County, California [3,4,5]. This is one of the most efficient potable water recycling facilities in the world that is providing promising solutions to many of the water supply or wastewater disposal challenges currently facing urban planners noted Ormerod [3]. In a recent publication, “Characterization of the Microbiome at the World’s Largest Potable Water Reuse Facility” by Stamps et al. [6], the authors reported a 5-log reduction in total bacterial load between source, unchlorinated wastewater feed, and the final effluent after treatments consisting of microfiltration, reverse osmosis, and ultraviolet/advanced oxidation. They also reported decreases in microbial diversity and load by several orders of magnitude after microfiltration and reverse osmosis treatment. Their findings reported levels of microbial composition to almost non-detectable levels that more closely resembled controls of molecular grade laboratory water than the biomass detected in the source water. One of the greatest results of this facility is the reduction of antibiotic resistance genes and viruses to almost undetectable levels. As noted above, the Orange County Water District (OCWD) Advanced Water Purification Facility (AWPF) is a highly engineered system designed to treat and produce up to 100 million gallons per day (MGD) of purified water from a municipal wastewater source for potable reuse.

The developments in modern methods of DNA sequencing such as next generation sequencing (NGS) and third generation sequencing (MinION) has greatly enhanced our abilities to detect other potentially pathogenic microorganisms that fail to grow using traditional cultivation media [7,8]. Also, sequencing of RNA, may detect active microorganisms in wastewater or biomass digestion systems [9] that could not be detected by traditional methods. Recently, microbial water quality and community analysis composition were summarized based on high-throughput sequencing for potable reuse [10]. Water reuse is essential and is recognized as an alternate source of water that is necessary in water-limited regions as part of a diverse water supply portfolio [6]. Therefore, these authors suggested that a comprehensive understanding of water quality from treatment, to tap, and throughout the reuse process is therefore paramount. As reported by Stamps et al. [6], prior to microfiltration and reverse osmosis filtration, the bacterial and archaeal community were not significantly different from the secondary treated wastewater. However, after treatments, the reverse osmosis membrane depleted the microbial biomass estimated by 16S rRNA gene quantitative PCR, but it also significantly reduced most detectable major ions. The reverse osmosis membrane was also the main barrier for the transmission antibiotic resistance genes (ARGs) that may otherwise be transmitted to other organisms in the environment once the water is used or discharged [11]. Stamps et al. [6] also showed that microfiltration membrane was the most effective region for the removal of ARGs. From the study the authors observed that no ARG data exists for water beyond the MF effluent. These authors noted that the system was so effective in the removal of microorganisms that they were unable to produce enough quantities of DNA for metagenomic sequencing even with large volumes of sampled water which was the original goal of their study. The findings from this study and other studies from OCWD AWPE [2,10,12,13] showed that advance waste water treatment has the potential to reduce microbial contaminants to undetectable levels. 

Numerous factors influence the quality of drinking water from large metropolitan areas to the smaller cities. However, one common factor is the water distribution systems which if not managed properly can result in some serious health effects as recently reported in Flint, Michigan, USA [14,15]. Between 2014 and 2015 there was a major outbreak of Legionnaires’ disease (LD) in Genesee County, Michigan, and this coincided with changes in the source of drinking water to Flint’s municipal water system [14]. Following the switch in water supply from Detroit to Flint River water, the odds of a Flint resident presenting with LD increased 6.3-fold (95% Cl: 2.5, 14.0) [14]. As the water source changed, there was exclusion of the anticorrosive agent, orthophosphate, from the Flint water-supply system that resulted in sustained damage to the municipal water system, leading to the leaching of toxic lead from pipes and fixtures into the municipal water [16]. During this period, outbreak of LD [17] was revealed in the summers of 2014 and 2015 in Genesee County, MI, where Flint is the largest town, with 87 cases of LD confirmed during the period of sustained damage to the municipal water system in Flint [16]. *Legionella pneumophila* infection is the leading cause of the disease due to drinking water in the United States [18]. Fresh water is the naturally habitat for this bacterium, but most LD originate in engineered water systems. Contaminated aerosols by devices such as cooling towers, hot tubs, whirlpools, decorative fountains, and showers that release water vapors, [14,19] are sometimes the main sources of infections to people.

## 2. Impact of Biofilms on Water Quality and Biostability

One of the factors causing persistence of this pathogen in the drinking water distribution systems (DWDS) is biofilm formation and may result in microbial contamination of drinking water. In fact, biofilms are the predominant mode of microbial growth in DWDS. Biofilms are often protected by extracellular polymeric substance (EPS) from environmental and shear stresses. Biofilms also present a significant problem to the drinking water industry as a potential source of bacterial colonization, including pathogens, and in many cases, also affecting the taste and odor of drinking water and promoting the corrosion of pipes [20]. Furthermore, biofilm formation on the metal surface in water distribution systems is associated with an important economic issue, i.e., increased risk of corrosion in these structures [21]. The first two steps in biofilm formation are conditioning of the material surface and the non-permanent binding of the cells to that surface. The next steps are the irreversible binding and the development of microcolonies. Finally, the biofilm’s three-dimensional structure is formed, giving rise to a complex ecosystem [22]. Biofilms consist of an EPS matrix, which is responsible for the integrity of the biofilm’s three-dimensional structure and is responsible for gluing cells together and onto surfaces. The EPS also provides protection for the microbial community from adverse environmental conditions [20]. Biofilm formation confers many advantages to the microbial cells in the distribution system such as physical, mechanical, and chemical protection [23].

Biofilm can harbor living and dead bacteria, protozoa and many other microflorae. Biofilm has a complex microbial community composition, and the bacteria must compete for the required nutrients to become an integrated member of the microbial community. Therefore, biofilm-associated bacteria must seek for the bacterial neighbors and the environment that best suits their growth and survival [24]. Bacteria are generally the dominant members of biofilm microbial communities in DWDS due to their high growth rates, relatively small size, adaptation capabilities, and ability to produce EPS [25]. Biofilm embedded microbes account for about 95% of the total biomass in DWDS, and use complex structures to protect themselves against antimicrobials, acquire new genetic traits, and metabolic activities for survival in a hostile environment [26]. Viruses, fungi, algae, and protozoa are also present, but at relatively smaller proportions [20].

One of the factors affecting water quality in DWDS is environmental fluctuations of the pipes. The presence of suspended and biofilm embedded microbes in DWDS have been shown to degrade the quality of distributed water for decades [26]. These authors investigated biofilm formation and water quality under various nutrients in DWDS, including chlorine concentrations and hydrodynamic conditions. They showed that turbidity variations, concentrations of total organic carbon, NH4^+^-N, and soluble phosphorus may induce changes in the DWDS environment resulting in microbial growth. They suggested that low nutrient availability and addition of mild chlorine at 0.50 mg/L at 0.50 m/s flow velocity were the most favorable conditions screened for optimized comprehensive performance, while nutrient supplements yielded significant performance deterioration. It has also been shown that elevated microbial growth and biofilm accumulation on DWDS pipes could shape the micro-environment of these pipes and cause severe water quality issues such as persistence of opportunistic pathogens, acceleration of pipe corrosion, as well as changes in color and odor [27]. In a study to determine the quality of reclaimed water in treated effluents in the distribution system of four plants in California, Florida, Massachusetts, and New York, with different treatment processes [28], indicator bacteria were detected in the effluent of only one system, but it was not detected at the sampling points, suggesting that its survival in the system was poor. These bacteria included heterotrophic bacteria, coliforms, *Escherichia coli*, enterococci, and pathogens, such as *Aeromonas* spp., *E. coli* O157:H7, *Legionella* spp., *Mycobacterium* spp., and *Pseudomonas* spp., as well as algae. Although all of the treatment systems effectively reduced the levels of bacteria in the effluent, bacteria regrew in the reservoir and distribution systems due to the loss of residual disinfectant coupled with high assimilable organic carbon (AOC) levels. AOC is readily available for consumption by microorganisms, which in turn can enhance the regrowth of bacteria in the reclaimed water. A strong positive correlation has been reported between *Mycobacterium* and AOC levels (17–234 mg/L) in potable water systems that had only a fraction of the AOC levels encountered in the reclaimed water systems [29]. Therefore, the loss of residual disinfectant coupled with the increase in AOC concentration in the systems may result in the increase in the level of bacteria, indicating that it is necessary to maintain a sufficient and stable residual level of disinfectant. The rapid dissipation of free chlorine may have been a result of its ability to react with organic matter, as reflected by the high organic carbon concentration compared to the concentrations typically encountered in potable water [28]. In a recent study that assessed LD outbreak in Flint, Michigan, Zahran et al. [14] reported that when water was drawn from the Flint River, free chlorine residual associated with mitigation of LD risk was nearly five times greater than it was before the switch in water supply (1.4 versus 0.3 mg/L). These authors concluded that their response model was indicative of an increase in free chlorine demand that was consistent with reports during the water crisis of enhanced levels of iron and assimilable organic matter, both of which promote legionellae growth as well as react chemically with free chlorine, thereby reducing its availability for disinfection reactions.

Environmental factors such as elevated flow velocity, chemical disinfectants, and carbon sources may impact microbial activities and water quality in DWDS. Inorganic nutrients such as nitrogen, phosphorus, and high organic matter contents may influence the quality of water in DWDS resulting in the formation of biofilms [30,31]. Phosphorus in the form of phosphate is added to water distribution systems to passivate metal surfaces by forming stable complexes with corroded surface metals [32] which limit further corrosion. Addition of phosphorus to DWDS has also been shown to result in changes in the biofilm structure and microbial community within the pipes [33]. These authors observed the formation of thicker, more-heterogeneous biofilms with a higher number of micro-colonies after phosphate treatment. Another environmental factor that may enhance biofilm formation in DWDS is nitrogen. Nitrogen is a building block for proteins and it is a key inorganic nutrient that is used by autotrophic nitrifying bacteria for biofilm development [34,35] in the presence of ammonia during chloramine decay [36]. Biological stability of water is conditioned by both the content of organic and non-organic substances. For instance, nitrogen, phosphorus, and organic substrates may create conditions for the growth of heterotrophic organisms, including pathogenic microorganisms in the DWDS [37,38,39]. It has also been shown that trace metals such as iron and copper can affect biofilm development in DWDS [40,41,42]. This may influence EPS formation as well as cell surface hydrophobicity [20] and has been observed in *L. monocytogenes* [43], *P. aeruginosa* [44], and other biofilm forming bacteria [45].

Flow velocity in DWDS may dramatically vary between different locations, alternating from laminar to turbulent flow and vice versa [20]. During the initial cell adhesion and biofilm formation stages, high flow rates can facilitate transport of bulk water microorganisms and their subsequent contact with surfaces as a result of convective diffusion [46] leading to a boost in EPS production and enhanced cell-to-substratum adhesion [43], resulting in mechanical stability of the growing biofilms. High flow rate also contributes to high nutrient transport rate from bulk water into the biofilm in DWDS thus stimulating additional growth [47]. Similarly, water velocity, shear force and laminar flow directly influence adherence of bacteria cells to surfaces. On the contrary, high flow rates can promote detachment of mature biofilms due to increased shear stress on the outer layers of the microbial communities resulting in poor quality of drinking water. Also, nutrient transport and shear effects are dampened at low flow rates [47] resulting in biofilms with loose structural integrity [42]. If DWDS are poorly designed, stagnation of water may occur and provide a suitable environment for biofilm formation. Potential adverse health effects may spread throughout the system. For example, stagnant water favors growth of biofilms, adherent microbial communities that are difficult to eradicate [48].

## 3. Microbial Composition in DWDS Biofilms Influencing Water Quality

Most guidelines on bacterial composition in DWDS and bacterial pathogens in wastewater and reclaimed water are based on the use of indicator microorganisms (e.g., *E. coli* and *Enterococci*) [49], as well as research utilizing culture-based methods analyzing single species of bacteria in nutrient rich environments. There are over 500 waterborne pathogens of potential concern in drinking waters [50,51,52,53,54,55,56,57,58,59,60,61,62,63,64,65,66,67,68], identified by the US Environmental Protection Agency (EPA) through its Candidate Contaminant List (‘CCL 3 Universe’ list, available at http://www.epa.gov/safewater/ccl/pdfs/report_ccl3_microbes_universe.pdf.). Table 1 summarizes infectious agents potentially present in untreated (raw) waste water. Due to current available technologies, these approaches do not provide a comprehensive analysis of microbial water quality since indicator microorganisms have been shown to be poorly correlated with the presence of pathogens in reclaimed water [28,68], and pathogens exist as members of complex microbial communities [51]. Culture-based techniques therefore underestimate most of the time the diversity and relative abundances of microorganisms in biofilms [69]. Although most state regulations require the use of chlorine residuals in water distribution systems, declines in the microbiological quality of water by the time it reaches end users have been previously documented [28]. Opportunistic pathogens (e.g., *Aeromonas* spp., *Mycobacterium* spp., and *Legionella* spp.) have been observed to regrow in disinfected water distribution systems due to biofilm development [70] and disinfectant dissipation [28] and have also been detected more often than routinely tested indicator microorganisms [28]. *E. coli* can grow on stainless steel, Teflon, glass, polystyrene, polypropylene, PVC, and biotic surfaces due to bacterial surface hydrophobicity, surface charge, and the expression of type 1 fimbriae [71]. These bacteria survive on dead bacterial tissue within the biofilm by deriving the required carbon, nitrogen, and amino acids for multiplication as well as from amoeba [49,72]. Amoeba can serve as a habitat that provides the environmental host for survival and replication of *Legionella* species in different environmental settings [73,74]. Various amoeba such as *Acanthamoeba castellanii* can use *L. pneumophila* as a sole food source [75], but amoeba also contribute to the spread of *L. pneumophila* and protect the bacteria from various adverse effects such as antibacterial agents [76]. *L. pneumophila* has also been shown to survive and replicate within protozoa that graze on biofilms [29]. High doses of disinfectants may be required to exterminate legionellae residing within protozoa or biofilms [77,78,79,80,81]. It has been reported that *L. pneumophila* can persist in complex engineered water systems and cause recurrent disease outbreaks for decades despite repeated efforts to eliminate them [82,83]. Due to persistence of *L. pneumophila* in biofilms, this pathogen was found in 70% of Pittsburgh and 60% of Paris hospital water systems [84,85]. The impact of water chemistry, pipe material, and stagnation on the building plumbing microbiome was examined in five water utilities across the U.S [86]. It was suggested that total chlorine concentration, pH, P, SO_4_^2−^, and Mg were associated with most of the variation in bulk water microbiome composition, and disinfectant type exerted a notably low-magnitude impact on microbiome composition. They also showed that WWTP with highest pH of 9–10 had the highest frequency of detection for *Legionella* spp. and lowest relative abundance of *Mycobacterium* spp. They confirmed that water quality at the tap varied based on location in the distribution system, even a 0.5 d water age can provide insight into the relative degrees of associated microbial shifts. They showed that water samples after stagnation yielded 6–13 more phyla compared to corresponding influents (before/after stagnation) likely due to “seeding” from building plumbing biofilm, or regrowth of rare species above the detection limit, likely as a result of disinfectant decay and the magnified influence of biofilms in the small diameter pipe [83]. Finally, premise plumbing pathogens of concern where enumerated by Pruden et al. [11] (Table 2). Other premise plumbing pathogens include *Stenotrophomonas maltophilia*, *Acinetobacter baumannii*, and *Sphinogmonas paucimobilis.*

Recent microbiome research is bringing new understanding to the true extent and diversity of microbes that inhabit water distribution systems. Schmeisser et al [87] found that most microbes in drinking water biofilms were closely related to *Proteobacteria*, and that *Proteobacteria* were the dominant organisms in biofilms formed on PVC, stainless steel, and cast-iron surfaces [88]. Twelve bacterial phyla, including members from *Nitrospirae*, *Acidobacteria*, and *Planctomycetes*, were detected in low assimilable organic carbon content (10 μg/L) DWDS pipes using 16S gene analysis, in comparison to the detection of only bacteria from the *Proteobacteria* and *Bacteriodetes* phyla using a cultivation-based method [89]. This technology has helped in more-thorough and -frequent monitoring of biofilm development and has become feasible for DWDS biofilm sampling and characterization. 

It has also been shown that source water and/or the water quality shaped by their respective treatment processes may play an important role in shaping the bacterial communities in the distribution system [90]. These authors studied biofilm communities using 16S rRNA gene clone libraries and functional potential analysis generated from total DNA extracted from coupons in biofilm annular reactors fed with onsite drinking water for up to 18 months. They observed differences between biofilm from ground water sources and surface water and suggested that the differences were associated with the classes *Beta Proteobacteria*, *Alpha proteobacteria*, *Actinobacteria*, *Gamma Proteobacteria*, and *Firmicutes*. Their study showed that after nine months the biofilm bacterial communities from both ground water and surface water were dominated by *Mycobacterium* species, and this positively correlated with DWDS temperature.

Another emerging pathogen in distribution systems is *L. pneumophila* [90]. This is an opportunistic waterborne pathogen and the causative agent for LD. This has been shown to be transmitted to humans via inhalation of contaminated water droplets. The bacterium can colonize a variety of man-made water systems such as cooling towers, spas, and dental lines and is widely distributed in multiple niches, including several species of protozoa as reported by Ashbolt [91]. The authors also noted that *L. pneumophila* can survive and persist within multi-species biofilms that cover surfaces within water systems. The main advantages that the biofilm confer on the pathogen were the ability to persist, spread, and resist treatments. In the United States, three plumbing-associated opportunistic pathogens (i.e., *Legionella pneumophila*, *Mycobacterium avium*, and *Pseudomonas aeruginosa*) are linked to cause ~41,000 infections per year, mostly affecting elderly populations and immunocompromised individuals [29,92]. 

One significant factor that adds drastic changes in bacterial cell counts and community composition in water distribution systems is water stagnation in buildings [93]. They observed a spatially structured bacterial community within the plumbing system following stagnation in building-impacted tap water and found it to be highly reproducible. They suggested that pipe diameter deterministically affected two ecologically relevant physical/chemical processes, namely the dispersal of bacteria from pipe surface biofilms and the decay of disinfectants. They showed that small-diameter pipes at the distal ends of building water supplies harbored the highest cell counts and deviated most from the city-water supply microbiome. This study highlights small-diameter pipes as a site for biological regrowth. The authors, however, suggested that the risk with small-pipe diameters cannot be managed by simply increasing the size of pipes. They indicated that larger diameter pipes could lead to increased levels of water stagnation and consequent water quality deterioration [86]. They suggested upgrading their end-point disinfection to counteract hypochlorite decay, address within-pipe cell growth and precise flushing of smaller-diameter pipes to prevent stagnation while minimizing water waste, and the use of biofilm-inhibitive materials in making small-diameter pipes. The relevance of water stagnation in the DWDS with reference to public health was recently evaluated in drinking water samples collected from homes receiving municipally treated drinking water with residence times of >24 h had higher concentrations of *M. avium* subsp. *avium* than drinking water from homes closer to the treatment plant [92]. This approach was applied to drinking water samples collected from 15 households serviced by a chloraminated distribution system, with homes located in areas representing short (<24 h) and long (>24 h) distribution system residence times [94]. Multivariate statistical analysis revealed that greater water age (i.e., combined distribution system residence time and home plumbing stagnation time) was associated with a greater relative abundance of *Mycobacterium avium* subsp. *avium*, one of the most prevalent non-tuberculous mycobacteria (NTM) causing infections in humans [95]. These authors noted that drinking water from homes closer to the treatment plant (with a shorter water age) contained more diverse NTM species, including *Mycobacterium abscessus* and *Mycobacterium chelonae*. It should be noted that only a few NTM are frequently detected in drinking water and drinking water distribution systems, including species commonly associated with human infections (e.g., *Mycobacterium avium* and *Mycobacterium abscessus*), as well as species that rarely cause infections (e.g., *Mycobacterium frederiksbergense* and *Mycobacterium aurum*) [96,97,98,99,100]. They concluded that drinking water from homes with residence times of >24 h had higher concentrations of *M. avium* subsp. *avium* than drinking water from homes closer to the treatment plant.

## 4. Prevention of Biofilm Formation in the Water Distribution System

There are many methods used for the prevention of biofilm formation in DWDS (Table 3). These include physical processes such as flushing, pigging, or air water scouring [14]. Chemical processes include chlorination and application of chloramines. Some biological processes target the quorum sensing process or EPS formation [26]. For many years, application of chlorine has been the main method used to limit the formation of biofilms in DWDSs. This is because it is cheap and efficacious and affects biofilm formation at every stage of development. Unfortunately, chlorine reduces microbial growth rate [101,102] and yet is incapable of the complete inhibition of biofilm growth [103] due to slow penetration of chlorine into biofilms. It should be noted that the use of chlorination may lead to the selection and increase of resistant bacteria, such as the opportunistic pathogen *M. avium*, and some unexpected increases in the number of opportunistic pathogens due to its relative resistance to chlorine [104], as well as enrichment of antibiotic resistant bacteria (ARB) in drinking water [105]. On the other hand, chloramines may maintain disinfection residuals for a longer period throughout the distribution system and generate fewer harmful regulated disinfection byproducts [104]. However, this compound is less reactive in comparison to chlorine, but may penetrate biofilms more effectively [106]. One great disadvantage using chloramines is the growth of certain nitrifying bacteria [104]. Another option is to coat pipe interiors with agents that block biofilm growth such as Sharklet, a synthetic material that mimics the rough texture of natural shark skin which resists biofouling and reduces biofilm formation by *M. avium* [107]. This novel coating has microscopic ribs that discourage pathogens from settling on it. Sharklet’s micro-patterned surface deters the formation of biofilms without the use of chemical antimicrobials. Materials such as this may be one way to keep bacteria from colonizing plumbing systems. Sharklet Technologies is currently evaluating the material’s ability to prevent biofilm fouling in surgical and hospital settings [108].

One of the most recent technologies for the control of biofilm growth is the use of nanotechnology agents [26]. Nanoparticles (NPs) are different from their bulk chemical counterparts because of their large surface area to volume ratio, which creates a higher number of functional sites and can enhance the influence of NPs on a given microorganism. Other methods for biofilm control also include strategies to reduce the levels of assimilable organic carbon (AOC), as well as the concentration of suspended microbes in drinking water prior to entering the distribution system (typically through membrane biofiltration). Nutrient limitations can also inhibit biofilm formation, induce biofilm dispersal, or both, ultimately reducing or delaying the impact of biofilms on engineered systems [26,43,109,110,111]. UV disinfection, oxidative treatments (ozonation), and a combination of UV and H_2_O_2_ treatment are other technologies that could also be used to reduce suspended microorganisms in drinking water prior to entering the distribution system, and these treatments may reduce chlorine demand and corrosion potential [112]. The goal is the limitation of corrosion of pipes in the distribution systems which will ultimately result in limiting biofilm growth because most corroded pipe surfaces are favorable over “smooth” surfaces for microbial attachment and colonization, and corrosion products have been known to promote the growth of unique biofilm-forming bacteria. In most systems, phosphate is added to prevent corrosion of pipes and this is a very inexpensive process, and more economical. 

The CDC, EPA, and Council of State and Territorial Epidemiologists (CSTE) have maintained the collaborative national Waterborne Disease and Outbreak Surveillance System (WBDOSS) to document waterborne disease outbreaks (WBDOs) reported by local, state, and territorial health departments since 1971. WBDOs were reclassified to better characterize water system deficiencies and risk factors, and the data were analyzed for trends in outbreak occurrence, etiologies, and deficiencies from 1971 to 2006 [113]. This document showed that a total of 833 WBDOs, 577,991 cases of illness, and 106 deaths were reported from 1971 to 2006.

## 5. Conclusions

The goal of water treatment is to provide potable clean water to the population. Therefore, availability of potable water devoid of pathogens is fundamental to public health. In advance countries this can be achieved with the right investment in water treatment infrastructure. However, in developing countries, where resources are limited, this at times may be very expensive for the government even at the federal level to achieve. The net effect is that the local people are exposed to poor water quality, exposing them to different opportunistic pathogens. This is because with poor water treatment systems, the population may end up with a combination of poor microbiological quality and poor water chemistry. The poor water chemistry in the main distribution lines will ultimately shape the microbiome in drinking water biofilms, thus enhancing the potential associations between opportunistic pathogens and indigenous drinking water microbes. Other factors such as disinfectant type and concentration, pipe material, and water age strongly influence the composition of the microbiome. However, specific effects of disinfectant, pipe material, and water age may vary in different distribution systems with various source water qualities. Disinfectants such as chlorine and chloramine may likely play role in shaping the microbiome biofilm. Chloramine may penetrate biofilm more strongly, while chlorine may better inactivate microorganisms near the biofilm surface resulting in the survival of microbes inside the biofilm. A modern technology in water distribution systems that may reduce the rate of biofilm formation is the use of biofilm-inhibitive materials in making small-diameter pipes that can coat pipe interiors with agents that block biofilm formation growth. Recently, nanotechnology agents has been introduced to some water treatment processes which creates a higher number of functional sites and can enhance the influence of NPs on a given microorganism. In all DWDSs, AOC concentration and other nutrients should be maintained at very low rates to inhibit the regrowth of bacteria. In general, it has been suggested that local environmental conditions are shaping biofilm formation, composition and amount, and hence managing these is critical for the best operation of DWDS to safeguard water quality [114]. Therefore, both qualitative and quantitative methods of biofilm control in the water distribution system must be one of the top priorities for any treatment facility. Public health is threatened by deteriorated water quality due to bacterial regrowth and uncontrolled growth-related problems in drinking water distribution systems and can lead to outbreak of water-borne illnesses. Therefore, the restriction of AOC concentration may be effective strategies to limit bacterial regrowth in DWDS. The final goal of water treatment is to achieve good quality drinking water at the taps of customers. Therefore, drinking water distribution system must act as a protective system to prevent contamination and bacterial regrowth as the treated water travels to the customer. This would accomplish the goal of The World Health Organization that states that ‘Water entering the distribution system must be microbiologically safe and ideally should also be biologically stable.’ Therefore, wastewater treatment facilities should continue to conduct multidisciplinary environmental surveillance and laboratory research to identify any risk in their systems so as to inform managements of future research and strategies for continuous availability of safe public water supplies. Such research may include work in nutrient management to reduce biofilm formation and maintain biological stability of the DWDS. Also, research into advanced processes to reduce biological contaminations such as bacteria, virus, protozoa, and other contaminants such as antimicrobials before the water is released into natural streams. Such may include additional work in reverse osmosis and microfiltration which are already available in many WWTPs. 

## Figures and Tables

**Table 1:** Infectious agents potentially present in untreated (raw) wastewater. From Environmental Protection Agency (EPA) [53], National Research Council (NRC) [54], Sagik et al. [55], World Health Organization (WHO) [56], Feachem et al. [57], Mara and Silva [58], Oragui et al. [59] Yates and Gerba, [60], da Silva et al. [61], Geldreich [62], Gerba [63], Haramoto et al. [64], Bitton [65], Blanch and Jofre, [66], and EPHC [67] as published in Rock et al [68]. The EPA Guidelines for Water Reuse summarizes infectious agents that may be present in untreated (raw) wastewater, reproduced here in Table 1 (EPA, [50]).

Pathogen	Disease	Quantity in Raw Wastewater (CFU/L)
*Shigella*	Shigellosis (bacillary dysentery)	Up to 10^4^
*Salmonella*	Salmonellosis, gastroenteritis (diarrhea, vomiting, fever), reactive arthritis, typhoid fever	Up to 10^5^
*Vibro cholera*	Cholera	Up to 10^5^
Enteropathogenic *Escherichia coli* (many other types of *E. coli* are not harmful)	Gastroenteritis and septicemia, hemolytic uremic syndrome (HUS)	
*Yersinia*	Yersiniosis, gastroenteritis, and septicemia	
*Leptospira*	Leptospirosis	
*Campylobacter*	Gastroenteritis, reactive arthritis, Guillain-Barré syndrome	Up to 10^4^
Atypical mycobacteria	Respiratory illness (hypersensitivity pneumonitis)	
*Legionella*	Respiratory illness (pneumonia, Pontiac fever)	
*Staphylococcus*	Skin, eye, ear infections, septicemia	
*Pseudomonas*	Skin, eye, ear infections	
*Helicobacter*	Chronic gastritis, ulcers, gastric cancer	
**Protozoa**		
*Entamoeba*	Amebiasis (amebic dysentery)	Up to 10^2^
*Giardia*	Giardiasis (gastroenteritis)	Up to 10^5^
*Cryptosporidium*	Cryptosporidiosis, diarrhea, fever	Up to 10^4^
Microsporidia	Diarrhea	
*Cyclospora*	Cyclosporiasis (diarrhea, bloating, fever, stomach cramps, and muscle aches)	
*Toxoplasma*	Toxoplasmosis	
**Helminths**		
*Ascaris*	Ascariasis (roundworm infection)	Up to 10^3^
*Ancylostoma*	Ancylostomiasis (hookworm infection)	Up to 10^3^
*Necator*	Necatoriasis (roundworm infection)	
*Ancylostoma*	Cutaneous larva migrams (hookworm infection)	
Strongyloides	Strongyloidiasis (threadworm infection)	
*Trichuris*	Trichuriasis (whipworm infection)	Up to 10^2^
*Taenia*	Taeniasis (tapeworm infection), neurocysticercosis	
*Enterobius*	Enterobiasis (pinwork infection)	
*Echinococcus*	Hydatidosis (tapeworm infection)	
**Viruses**		
Picornaviruses (including Aichi virus)	Gastroenteritis	
Enteroviruses (polio, echo, coxsackie, new enteroviruses, serotype 68 to 71)	Gastroenteritis, heart anomalies, meningitis, respiratory illness, nervous disorders, others	Up to 10^6^
Hepatitis A and E virus	Infectious hepatitis	
Adenovirus	Respiratory disease, eye infections, gastroenteritis (serotype 40 and 41)	Up to 10^6^
Rotavirus	Gastroenteritis	Up to 10^5^
Parvovirus	Gastroenteritis	
Astrovirus	Gastroenteritis	
Caliciviruses (including Norovirus and Sapovirus)	Gastroenteritis	Up to 10^9^
Coronavirus Gastroenteritis	Coronavirus Gastroenteritis	

**Table 2:** Premise Plumbing Pathogens of Concern: Diseases, Cases, Modes of Exposure, and Regulations (CCL—Contaminant Candidate List) from Pruden et al. [11].

Pathogen	Disease(s)	Cases/Deaths (Year(s))	Mode of Exposure	Reportable	Regulations
*L. pneumophila*	Legionnaires’ disease (pneumonia) or Pontiac fever	4107 U.S. cases	Inhalation or aspiration	Yes	Yes
*M. avium*	Pulmonary disease, cervical lymphadenitis (children)	19,600 U.S. cases	Inhalation or aspiration	No	No, but listed on CCL3
*P. aeruginosa*	Urinary tract infections, respiratory infections, dermatitis, soft tissue infections, bacteremia, bone and joint infections, GI infections	1400 U.S. pneumonia deaths, 2.4 million U.S. ear cases	Wound infection; inhalation	No	No
*Acanthamoeba*	*Acanthamoeba* keratitis (AK) Granulomatous amoebic encephalitis (GAE)	>3000 global cases	Wound infection; contact lens solution	No	No

**Table 3:** Engineering approaches to control opportunistic premise plumbing pathogens (OPPPs). From Pruden et al. [11].

Technique	Advantage	Limitations/Disadvantage
Strategies Applied in Individual Buildings		
Maintain > 60° C in all hot water lines	Highly effective in reducing hot water risks for all OPPPs	Rapid scaling in some waters, higher energy losses, higher scalding potential. Not currently recommended in U.S. for individual residences.
Temporarily increase temperature > 60 °C	Briefly effective	*Legionella* growth sometimes rampant after temperature is decreased. Cannot be recommended currently.
Dosing of Chloramine and chlorine dioxide in building	Reported highly effective in institutional applications	Concern regarding chlorine corrosion of copper, brass, stainless steel and plastic plumbing. Potential increase in predominance of *M. avium.* Chlorine dioxide corrosion effects uncertain.
UV-radiation	Kills/removes bacteria	Regrowth of OPPPS at all points downstream of devices and potentially within devices themselves.
Copper/silver ionization	Some benefits reported in field studies; efficacy data needed	Requires maintenance, *M. avium* complex are Ag and Cu resistant and predominance might increase. Potential for deposition corrosion.
Point of use filters	Quick connection	Has the potential to harbor bacteria, concentrate nutrients, remove disinfectants, and foster growth of pathogens downstream. Shower type filters are not yet proven but could be attractive.
Plumbing material	e.g., inhibitory effect from copper pipes if Cu^2+^ high enough	Effects certainly not universal, and could be difficult to control, and may be in conflict with aesthetic and EPA LCR goals.
Prevent stagnation	Belief that flow pattern is important	Unproven. Direct experimentation actually indicatesLegionella incidence was increased by continuous flow.
Avoid metered faucets	Deemed influential for *P. Aeruginosa* and *Legionella*	While cause and effect has not been clearly established, a growing body of evidence suggests serious problems with these devices.
Community Based Responses (i.e., could be applied at treatment plant by utilities)		
Chloramine	Very effective control of *Legionella* when residual is present	Chloramines can be low or absent in premise plumbing, especially in “green” construction, if nitrification is occurring, or at ends of system. Possible increased incidence of *M. avium*
Remove AOC/BDOC at treatment plant	Focus of WRF project #4251	Recent work in Netherlands and in project #4251 strongly suggests it will not be an effective community based response by itself.
Maintain Distribution System Pipes/Corrosion Control	Decreased biofilms and increased disinfectant.	Complex ecology suggests there sometimes could be merit to this approach, but would be costly and the benefits are not clear.
